# TRPV4 Channel Modulators as Potential Drug Candidates for Cystic Fibrosis

**DOI:** 10.3390/ijms251910551

**Published:** 2024-09-30

**Authors:** Razan Orfali, Ali AlFaiz, Madhawi Alanazi, Rahaf Alabdulsalam, Meaad Alharbi, Yara Alromaih, Ismail Dallak, Marah Alrahal, Abdulaziz Alwatban, Reem Saud

**Affiliations:** 1 Research Center, King Fahad Medical City, Riyadh Second Health Cluster, Riyadh 12231, Saudi Arabiameaad_6@hotmail.com (M.A.);; 2 King Abdulaziz Medical City, Jeddah 9515, Saudi Arabia; 3 College of Medicine, Imam Mohammad Ibn Saud Islamic University, Riyadh 13317, Saudi Arabia; 4 General Education Department, Dar Al-Hikmah University, Jeddah 22246, Saudi Arabia

**Keywords:** TRPV4 channels, lungs, motile cilia, cystic fibrosis, mucociliary clearance

## Abstract

Cystic fibrosis (CF) is a genetic disorder caused by mutations in the cystic fibrosis transmembrane conductance regulator (*CFTR*) gene, resulting in defective chloride ion channels. This leads to thick, dehydrated mucus that severely disrupts mucociliary clearance in the respiratory system and triggers infection that eventually is the cause of death of CF patients. Current therapeutic strategies primarily focus on restoring CFTR function, blocking epithelial sodium channels to prevent mucus dehydration, or directly targeting mucus to reduce its viscosity. Among the ion channels expressed in ciliated bronchial epithelial cells, the transient receptor potential vanilloid 4 (TRPV4) channel emerges as a significant channel in CF pathogenesis. Activation of TRPV4 channels affects the regulation of airway surface liquid by modulating sodium absorption and intracellular calcium levels, which indirectly influences CFTR activity. TRPV4 is also involved in the regulatory volume decrease (RVD) process and enhances inflammatory responses in CF patients. Here, we combine current findings on TRPV4 channel modulation as a promising therapeutic approach for CF. Although limited studies have directly explored TRPV4 in CF, emerging evidence indicates that TRPV4 activation can significantly impact key pathological processes in the disease. Further investigation into TRPV4 modulators could lead to innovative treatments that alleviate severe respiratory complications and improve outcomes for CF patients.

## 1. Introduction

**Figure 1:**
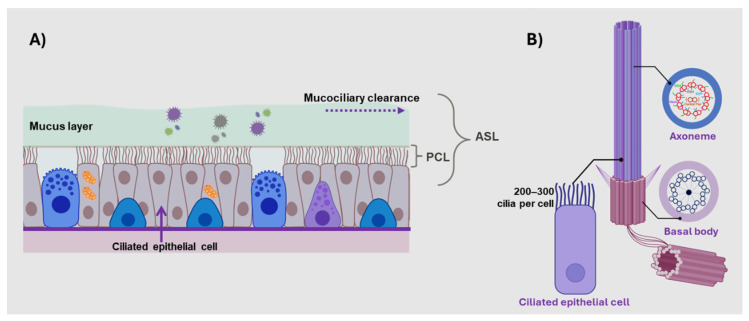
Mucociliary clearance system. (**A**) Cross-section of the differentiated conducting airway epithelium, illustrating ciliated columnar epithelial cells and secretory goblet cells (in blue with secretory granules). The apical airway surface liquid (ASL) is a low-viscosity periciliary layer (PCL) that enables effective ciliary beating. Above this, a more viscous mucus layer functions to entrap inhaled pathogens, providing a protective barrier for the respiratory tract. (**B**) Schematic diagram of a ciliary axoneme and basal body. Motile cilia have a 9 + 2 microtubule pair ultrastructure, with inner and outer dynein arms. The dynein arms are crucial for ciliary motility.

The human airway is protected by an advanced defense system that maintains lung sterility. Inhaled particles are consistently cleared from the airways via mucociliary clearance (MCC), which is crucial for respiratory health [1]. The MCC system comprises two primary components: mucus secreted by goblet cells and submucosal glands, which trap microbes and debris, and tiny hair-like motile cilia that line the airways and beat in a continuous rhythmic motion. The apical airway surface is lined by a complex airway surface liquid (ASL), consisting of two distinct layers. The upper gel-like mucus layer traps inhaled pathogens, while the underlying less viscous periciliary liquid (PCL) lubricates the airway surface, enabling the cilia to beat efficiently (Figure 1A) [2,3]. Adenosine triphosphate (ATP) is a vital energy source for ciliary motion. Motile cilia are primarily found on the ciliated epithelium lining the respiratory tract, brain ventricles, fallopian tubes, and spinal cord [3,4]. Respiratory cilia are highly specialized hair-like structures primarily composed of microtubule-based organelles originating from basal bodies on the apical membranes of airway epithelial cells [3,5]. Each ciliated airway epithelial cell contains approximately 200–300 cilia.

The structure of a motile cilium includes a microtubule-based axoneme with two single microtubules at the center, surrounded by nine peripheral doublet microtubules, forming a “9 + 2” arrangement. Each outer microtubule pair is equipped with inner and outer dynein arms, which produce the required energy for motility through ATP hydrolysis [3,4,6] (Figure 1B). Motile cilia beat in a coordinated metachronous pattern, facilitating the movement of inhaled debris trapped in the mucus layer out of the airways [7]. The cilium moves forward quickly and forcefully in a power stroke to propel the mucus gel layer. This is followed by a slower recovery phase, during which the cilium bends backward at a 90° angle, moving along the same plane to return to its initial position [2,3]. Defects in ciliary function can severely hinder MCC, leading to various airway diseases such as cystic fibrosis, a classic example of a respiratory disorder resulting from defective MCC [7].

The TRPV4 cation channel, a member of the transient receptor potential (TRP) vanilloid subfamily, plays a crucial role in cell volume homeostasis, medium viscosity, and the regulation of ciliary beat frequency (CBF) in epithelial cells [8,9]. Activated by various stimuli such as heat, mechanical forces, hypo-osmolarity, and arachidonic acid metabolites, TRPV4 integrates multiple environmental signals through its diverse regulatory sites [10]. TRPV4 has demonstrated high druggability, with various chemotypes yielding potent ligands that exhibit oral bioavailability and favorable drug-like characteristics [11]. This channel is essential for the physiology of ciliated epithelia and has been implicated in modulating respiratory function. Understanding the signaling mechanisms that govern TRPV4 function is vital, as it holds potential as a therapeutic target for respiratory diseases, including cystic fibrosis, the most prevalent muco-obstructive disease [12,13].

## 2. Cystic Fibrosis and CFTR Dysfunction

Cystic fibrosis (CF) is an inherited condition caused by mutations in the cystic fibrosis transmembrane conductance regulator (*CFTR*) gene. Over 2000 CFTR mutations have been identified, leading to dysregulated ion transport characterized by increased sodium absorption and reduced chloride and bicarbonate secretion into the ASL [14]. This imbalance decreases the water content in both the mucus and periciliary layers, producing thick, sticky mucus that impairs ciliary function [15]. Although infants with CF are born with seemingly normal lungs, they progressively develop chronic lung disease due to impaired mucociliary clearance, resulting in recurrent infections and bronchiectasis [16,17]. Respiratory failure remains the leading cause of mortality in individuals with cystic fibrosis [18]. Cystic fibrosis was considered a fatal childhood disease, with limited treatment options and an inappropriate prognosis [19].

### 2.1. Advancements in CF Clinical Care

Recent advancements in clinical care have been diverse and impactful. These include earlier diagnosis through the implementation of newborn screening programs, the standardization of airway clearance therapies, and the reduction of malnutrition through effective pancreatic enzyme replacement and a high-energy, high-protein diet [16,20]. In high-income countries, center-based care has become the standard, enabling patients to benefit from the expertise of multidisciplinary teams [21]. Pharmacological interventions now address respiratory symptoms by targeting airway mucus and surface liquid hydration alongside antimicrobial therapies such as antibiotic eradication treatments for early-stage infections and maintenance protocols for chronic infections [15]. Various CFTR modulator compounds and combinations are progressing through the preclinical to clinical development stages [22]. Therefore, dependable screening tools and personalized medicine approaches that can accurately predict drug efficacy are crucial to support translational research and develop individualized treatment plans [14]. Using nasal and bronchial epithelial cultures from individual CF patients for drug testing through in vitro assays, such as electrophysiological measurements of CFTR activity and assessments of ion and fluid movement in organoid cultures, allows for the prediction of patient-specific responses [22]. These patient-derived model systems offer valuable opportunities to forecast drug responses in individual CF patients and are expected to play a key role in advancing precision medicine for this population [14,20,22]. However, the exact significance and accuracy of these models in predicting long-term clinical outcomes have yet to be fully determined. Additionally, despite recent progress with CFTR modulators for cystic fibrosis, the continued development of new mucolytic, anti-inflammatory, and anti-infective therapies remains crucial, particularly for patients with advanced stages of lung disease [20].

### 2.2. Mechanisms of Mucus Dehydration and Impaired Mucociliary Clearance

**Figure 2:**
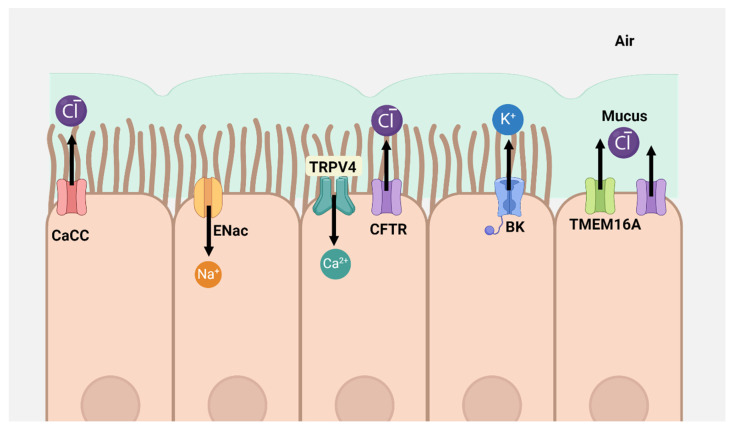
Simplified model of epithelial cells showing the organization of apical ion channels and receptors. TRPV4: transient receptor potential vanilloid 4 is expressed in ciliated cells, where it functions as a mechanosensory and chemosensory channel [23,27]. CaCC: Ca^2+^-dependent Cl^−^ channel, ENac: epithelial sodium channels, BK: big conductance Ca^2+^ activated K^+^ channels, TMEM16A: Ca^2+^ activated Cl^−^ channels. CFTR: cystic fibrosis transmembrane conductance regulator.

A critical function of airway epithelia is to regulate the fluid layer’s volume, pH, and viscosity to ensure normal lung function. This regulation requires the coordinated transport of solutes, ions, and water across airway epithelial cells’ basal and apical surfaces. The apical membrane contains various ion channels, including CFTR, that facilitate transepithelial chloride transport [17,23]. Mutations impairing CFTR function significantly disrupt ion and water transport, causing a range of harmful effects across various organs. The reduced secretion of chloride and bicarbonate in the lungs has several serious consequences [24]. Firstly, the decrease in electrolyte and water secretion results in the dehydration of airway surfaces, which hinders mucociliary clearance [25]. This problem is exacerbated by the increased activity of the epithelial sodium channel (ENaC) in CF airways, a condition partially linked to CFTR malfunction [24]. Secondly, diminished bicarbonate secretion leads to the acidification of the apical surface fluid, which compromises the effectiveness of antibacterial defenses [26]. Thirdly, the reduced bicarbonate secretion impairs the release and expansion of mucins from goblet cells and submucosal glands [24,26]. Although the importance of these alterations is not fully understood, the overall outcome is that the airways of CF patients become obstructed with thick mucus and are colonized by opportunistic bacteria like Pseudomonas aeruginosa, triggering a severe inflammatory response. This ultimately leads to progressive, irreversible structural lung damage and a decline in lung function [15]. In addition to CFTR, airway epithelial cells possess a range of ion, solute, and water transporters that are differentially distributed along the apicobasal axis. Channels for sodium, chloride, calcium, and potassium play essential roles in maintaining lung homeostasis (Figure 2) [23,27]. The importance of these channels is underlined by the fact that disruptions in epithelial ion transport, which alter fluid composition, are implicated in various human diseases [23].

More than two decades ago, functional studies uncovered that airway epithelial cells possess an additional mechanism for chloride secretion that operates independently of CFTR [28]. When cells were stimulated with calcium agonists such as UTP or ATP, both in vitro and in vivo (using nasal epithelia), there was a significant increase in chloride transport. This response was believed to be due to the calcium-dependent activation of a chloride channel distinct from CFTR, as it was also observed in patients with cystic fibrosis [29]. The discovery of this alternative chloride channel in airway epithelial cells led to clinical studies exploring the stimulation of calcium-dependent chloride secretion through the aerosol administration of denufosol [28]. Potassium channels also play a crucial role in various essential lung functions, including oxygen sensing, inflammatory responses, chloride transport enhancement, and respiratory epithelium repair [30]. The basolateral potassium channel also regulates sodium absorption, and a reduction in sodium absorption in the lungs has been associated with improvements in muco-obstructive diseases. These ion channels and transporters are crucial for transepithelial ion transport and play significant roles in lung function. This includes members of the TRP family [27,31,32].

## 3. TRP Channels in Cystic Fibrosis

**Figure 3:**
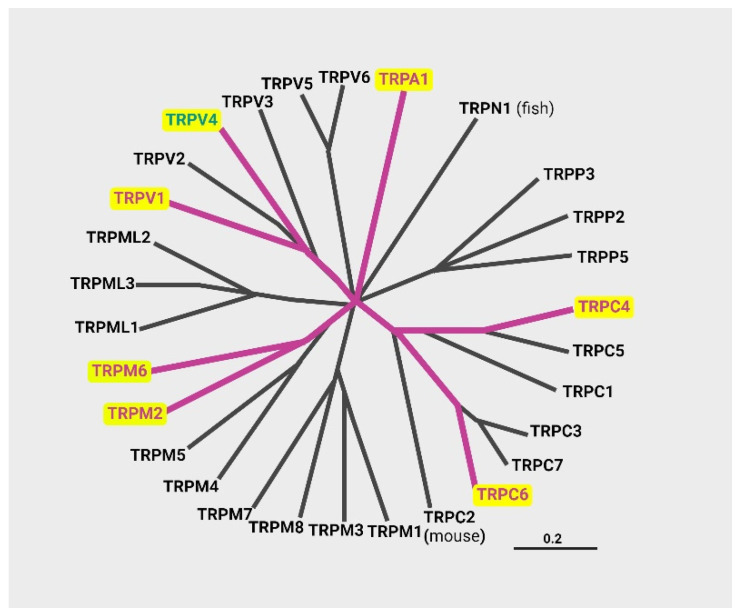
Phylogenetic tree of the transient receptor potential (TRP) channel family. A total of 28 human TRP channels have been identified to date. The TRP channels that are most prominently expressed in lung tissues are highlighted.

The transient receptor potential (TRP) multigene superfamily channels were originally discovered in Drosophila as phototransduction proteins. This action led to the identification of a large family of proteins (Figure 3) [33,34]. There are 28 mammalian TRP channels, which are divided into seven subfamilies: TRPA (ankyrin), TRPM (melastatin), TRPC (canonical), TRPV (vanilloid), TRPP (polycystin), TRPML (mucolipin), and TRPN (NOMPC-like). The latter is found only in invertebrates and fish [17,35,36].

TRP channels are involved in a variety of sensory responses, such as heat, cold, pain, stress, vision, and taste, and can be triggered by numerous stimuli. Their primary presence on the cell surface, interaction with various physiological signaling pathways, and distinctive structure make TRP channels probable for drug targets. This involvement suggests their potential to treat a broad spectrum of diseases [37]. TRP ion channels are broadly expressed in various tissues and cell types and are involved in diverse physiological processes, such as the sensation of different stimuli or ion homeostasis [38]. TRP channels are nonselective cation channels, only a few are highly Ca^2+^-selective, and some are permeable for Mg^2+^ ions. The TRP channel family can be activated by different mechanisms, from ligand binding, voltage, and changes in temperature to covalent modifications of nucleophilic residues [34]. When TRP channels are activated, they cause the cellular membrane to depolarize, subsequently triggering voltage-dependent ion channels and alterations in intracellular Ca^2+^ levels. Consequently, TRP channels are essential for functioning in intracellular organelles such as endosomes and lysosomes [39]. TRP gene mutation has been linked to various pathological states, including pain, skeletal dysplasia, lung diseases, and neurodegenerative disorders [40,41]. Targeting the TRP channels may offer new therapeutic approaches for related diseases [28].

In lung diseases, TRPA1, TRPC4, TRPC6, TRPM2, TRPM8, TRPV1, and TRPV4 are the TRP channels most abundantly expressed in lung tissues (Figure 3) [36]. TRP channels play a crucial role in maintaining cellular ion homeostasis, particularly for Ca^2+^ and Na^+^ that are vital for various functions in respiratory tract cells. Additionally, TRP channels may help detect and defend against harmful substances in inhaled air [42]. Many TRP channels are present in immune cells, where they regulate functions such as cytokine expression, migration, and phagocytic activity [12]. In the epithelial layer, the expression of TRP channels is significant in the pathogenesis of inflammatory disorders, primarily through the control of chemokine and cytokine expression and release. TRPA1 channels, for example, were found to modulate the inflammatory response in CF bronchial epithelia exposed to planktonic bacteria or mucopurulent material, mimicking *P. aeruginosa* infection [12,43]. TRPC6 channels in respiratory diseases are also involved in various cell types, such as macrophages, neutrophils, and smooth muscle cells. In CF human airway epithelial cells, TRPC6-mediated Ca^2+^ influx was significantly increased compared to non-CF cells when exposed to a diacylglycerol analog that activates TRPC6, highlighting the significance of these channels in CF [44].

## 4. TRPV4 Channel’s Structure and Functions

The TRPV4 cation channel, a member of the transient receptor potential (TRP) vanilloid subfamily, plays a crucial role in cell volume homeostasis [45], medium viscosity regulation [46], and the modulation of ciliary beat frequency [47]. The TRPV family, involving TRPV1 through TRPV6, consists of polymodal channels that can sense thermal, acidic, mechanical stress, and osmotic stimuli (Table 1) [48]. TRPV4 channels respond to various stimuli and are activated by heat, mechanical stimuli, hypo-osmolarity, and arachidonic acid metabolites [49]. Activation of TRPV4 increases intracellular calcium levels, which enhances ciliary beat frequency [50]. This mechanism can help alleviate obstructive pulmonary diseases, highlighting the potential of TRPV4 as a therapeutic target in cystic fibrosis [10].

### 4.1. Molecular Structure of TRPV4

**Figure 4:**
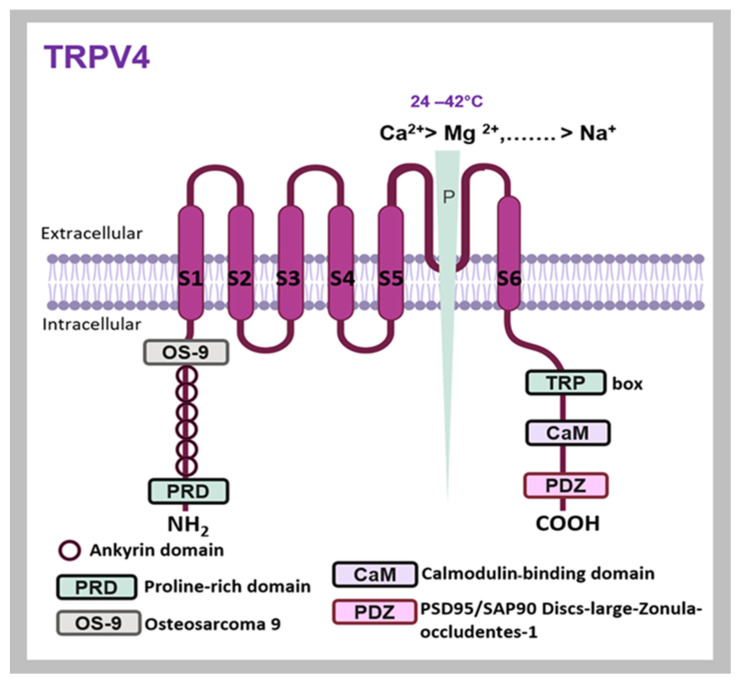
TRPV4 channel subunit. The N-terminus of TRPV4 includes six ankyrin repeat domains and a proline-rich domain (PRD), while the C-terminus contains calmodulin-binding domains and a PDZ domain. TRPV4 is responsive to temperatures ranging from 24 to 42 °C, with permeability following the sequence Ca^2+^ > Mg^2+^ > K^+^  ≈  Cs^+^  ≈  Rb^+^ > Na^+^ > Li^+^.

**Figure 5:**
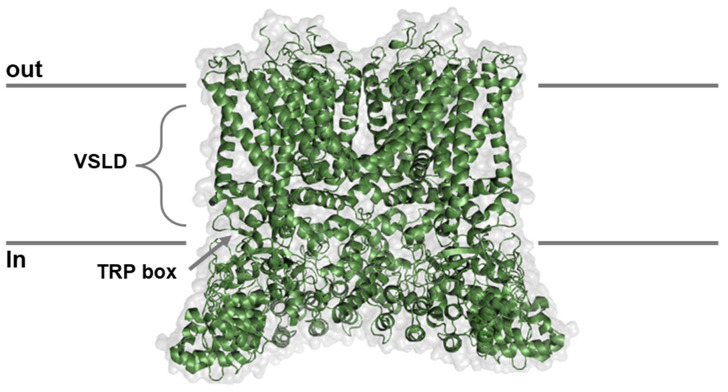
**Full-length human TRPV4 cryo-EM structure in the open state (PDB: 8T1D).** The four subunits of hTRPV4 are shown in green with views of the top (extracellular) layer and the bottom (intracellular) layer.

Structural insights gained from cryo-EM and X-ray studies have significantly enhanced our understanding of TRPV4 channel mechanisms [63]. In the human TRPV4 homotetramer, each subunit consists of 871 residues and is organized into two distinct layers [64]. The bottom layer, known as the cytoplasmic layer, includes the protein’s amino- and carboxyl-terminal regions [63]. The top layer, or transmembrane region, consists of six helices (Figure 4). The first four α-helices (S1–S4) form a voltage sensor-like domain (VSLD), similar to those found in other tetrameric voltage-gated ion channels (VGICs) [65], while the cation-permeable pore is located between S5 and S6 [49]. Figure 4 depicts a subunit of the TRPV4 channel, highlighting key structural features [66]. The N-terminus comprises six ankyrin repeat domains and a proline-rich domain (PRD) that is vital for the channel’s mechanosensitive functions [67]. The C-terminus includes calmodulin-binding domains necessary for Ca^2+^-dependent activation and a PDZ domain (PSD95/SAP90-Discs-large-Zonula-occludentes-1), which likely facilitates interactions with PDZ-domain proteins. TRPV4 is activated within a temperature range of 24 to 42 °C [68] and functions as a nonselective cation channel, exhibiting a permeability sequence of Ca^2+^ > Mg^2+^ > K^+^ ≈ Cs^+^ ≈ Rb^+^ > Na^+^ > Li^+^ [13]. The cryo-electron microscopy (cryo-EM) structures [69] of human transient receptor potential vanilloid 4 (hTRPV4) have provided valuable insights into the architecture and function of this ion channel (Figure 5). Co-crystallization and crystal soaking techniques have been employed to examine TRPV4 in complexes with various ions, including Cs^+^ and Ba^2+^ [63]. These studies have highlighted the structural features of TRPV channels, demonstrating the importance of using complementary methods to explore ligand interactions [70]. Comparisons between the cryo-EM structures of TRPV4 and other TRP channels have revealed both similarities and unique features [71]. Cryo-EM has emerged as a powerful technique for high-resolution visualization of TRPV4 channels, offering valuable insights into ion permeation, ligand binding, and gating mechanisms, which will be further explained in the following sections.

### 4.2. Physiological Roles of TRPV4 in Respiratory Epithelium

Over the past two decades, extensive research has been conducted on TRPV4 channels across a variety of diseases, with a particular emphasis on their role in the respiratory system. TRPV4 has emerged as a promising therapeutic target, offering significant potential for the development of novel treatments [72]. TRPV4 is an ion channel essential for several physiological processes, including the maintenance of the alveoli–capillary barrier [72,73,74], osmolarity sensing [75], chronic rhinosinusitis, thermoregulation [76], and mechanosensation in the vascular endothelium [72]. Its expression is notably upregulated in severe respiratory and cardiovascular conditions such as pulmonary hypertension, cystic fibrosis, asthma, and chronic obstructive pulmonary disease (COPD) [77]. TRPV4 is expressed in various lung tissues, such as pulmonary artery smooth muscle cells (PASMCs) [78], vascular endothelium, tracheal airway epithelial cells [79], and bronchial epithelial cilia [80]. TRPV4 plays a vital role in controlling pulmonary blood flow, ensuring fluid balance, and modulating immune responses, all of which are crucial for proper respiratory function [72]. The activation of TRPV4 is crucial in regulating respiration, primarily through the indirect stimulation of bronchopulmonary sensory neurons [81]. Research also suggests that TRPV4 channels play a key role in maintaining the lung alveoli–capillary barrier, which is linked to lung edema and injury. This barrier is essential for gas exchange, controlling permeability, clearing fluids, and protecting against infections [77]. This can highlight the critical role of TRPV4 in respiratory physiology and its potential as a therapeutic target in the treatment of lung diseases (see Table 2).

TRPV4 serves multiple physiological roles in the respiratory epithelium, including TRPV4 regulating calcium levels in respiratory epithelial cells and impacting key processes like ciliary beat frequency, which is crucial for clearing mucus and debris from the airways [50,85]. Its activation also promotes nitric oxide (NO) production in response to bacterial lipopolysaccharides (LPSs), which helps reduce inflammation and supports bronchodilation [86,88]. TRPV4 is vital for maintaining the alveolar epithelial barrier, preventing lung edema, and is involved in inflammatory signaling pathways.

These functions highlight the importance of TRPV4 in respiratory health and its potential as a therapeutic target in various pulmonary diseases.

## 5. TRPV4 Modulation in CF

Cystic fibrosis, the most common inherited disease affecting respiratory cilia, is caused by mutations in the CFTR gene, leading to impaired electrolyte balance and severe respiratory complications, including mucociliary dysfunction and eventually respiratory failure [15,89,90]. Despite the critical role of cilia in CF pathology, molecular studies of ciliated cells in human airways remain limited [17]. CF is characterized by impaired mucociliary clearance due to thick, dehydrated mucus that cilia cannot effectively clear from the airways, leading to recurrent infections and, eventually, respiratory failure [15,91]. This pathology results from the dysfunction of the CFTR chloride channel, which disrupts ion balance by reducing Cl^-^ secretion and increasing Na^+^ absorption through the epithelial sodium channel (ENaC), resulting in the dehydration of the ASL and production of viscous mucus [17,90,92]. Multiple studies have explored potential therapeutic strategies for CF, including restoring CFTR function [93], blocking ENaC [94,95,96], and reducing mucus viscosity [97]. Here, we present the various effects of TRPV4 modulation in CF.

### 5.1. TRPV4 and CBF Regulations

TRPV4 channels, expressed in the ciliated respiratory epithelium, are particularly important in regulating CBF, a key factor in mucociliary transport [98]. In CF epithelial cells, TRPV4-dependent calcium influx is reduced in response to hypotonic conditions [84,99]. Although airway surface dehydration is the primary cause of impaired mucociliary clearance in CF, enhancing CBF via TRPV4 activation is emerging as a potential therapeutic strategy [79,88]. Notably, tracheal epithelial cells lacking TRPV4 show decreased CBF in response to specific stimuli, suggesting that TRPV4 activation may improve mucociliary clearance [80]. However, its impact on CF outcomes requires further investigation.

### 5.2. TRPV4’s Role in Restoring the Regulatory Volume Decrease (RVD) Process

Regulation of cell volume is critical for maintaining cellular homeostasis. In hypotonic environments, cells swell due to osmosis, but many vertebrate cells counter this through a process known as regulatory volume decrease (RVD). TRPV4 may be involved in osmoregulation by responding to hypotonic stimuli with Ca^2+^ conductance. Becker et al. have shown that TRPV4 activation can restore the regulatory volume decrease (RVD) process [45], which is compromised in CF airways. To investigate TRPV4’s role in volume regulation, a TRPV4-EGFP fusion protein was expressed in CHO cells that normally lack TRPV4 and cannot undergo RVD in hypotonic conditions [45]. Fluorescence imaging confirmed that TRPV4 localized to the cell membrane. Expression of TRPV4 enabled CHO cells to perform typical RVD following hypo-osmolarity-induced swelling. This RVD response was significantly reduced by in a Ca^2+^-free environment, indicating that TRPV4 directly contributes to RVD [45].

### 5.3. TRPV4 Modulates Inflammatory Responses in CF

TRPV4 activation has been linked to the modulation of inflammatory responses in CF, potentially contributing to both therapeutic effects and inflammatory complications [43,84]. Using both cellular and animal models, Henry et al. found that epithelial TRPV4 plays a key role in triggering the production of major proinflammatory mediators and recruiting neutrophils in lung tissues [100]. Additionally, they discovered that TRPV4-dependent signaling is disrupted in a CF environment. TRPV4 activation in epithelial cells triggers the secretion of proinflammatory cytokines and lipid mediators, such as IL-8 and prostaglandin E2, particularly in response to bacterial endotoxins [100,101]. This inflammatory response is further increased when CFTR function is inhibited, highlighting the complex role of TRPV4 in CF pathology [84].

### 5.4. TRPV4 Activates CFTR Channels in CF Airway Epithelia

TRPV4 channels are found in the airways, specifically in smooth muscles, the alveolar wall, lung tissue, and lungs. TRPV4 channels respond to stimuli such as moderate heat, hypotonic stress, phorbol esters, and arachidonic acid metabolites, increasing intracellular calcium levels and influencing mucociliary function and inflammation [102,103,104]. TRPV4 stimulation increased intracellular Ca^2+^, which activated CFTR channels in bronchial epithelial cells under normal physiological conditions [105]. 4α-phorbol esters have been demonstrated to activate TRPV4, leading to increases in intracellular Ca^2+^ levels, which in turn affects the activity of other ion channels, including CFTR [72,99]. This interaction between TRPV4 and CFTR is significant in various physiological processes, particularly in epithelial cells, where the regulation of Na^+^ and Cl^-^ transport is crucial [23,103]. It was also found that activating the TRPV4 by GSK1016790A produced concentration-dependent calcium responses in TRPV4-expressing HEK293 and 16HBE cells, and the TRPV4 antagonist HC067047 caused a rightward shift of the GSK1016790A concentration–response curves [50]. These findings suggest that TRPV4 channels play a crucial role in regulating intracellular calcium levels, which in turn modulates CFTR channel activity in airway epithelial cells. This mechanism could have significant implications for understanding and potentially treating cystic fibrosis.

## 6. Pharmacological Modulators of TRPV4

Over the past decade, TRPV4 has emerged as a promising pharmacological target, with significant progress in developing potent and selective modulators. Understanding the druggability of TRPV4 channels and their ligands is essential for developing therapies in various medical fields such as oncology, cardiovascular diseases, and respiratory diseases. Although TRPV4 shows promising therapeutic potential, its druggability depends on the development of specific and effective ligands [41]. While biophysical and biochemical studies have advanced our understanding of TRPV4’s gating mechanisms and modulators [11,106,107], more structural studies are needed to clarify modulator binding sites, mutations, and functional changes [108]. Recently, a human TRPV4 structure has shown that certain compounds bind in a common region, the vanilloid pocket, located within a cytosol-facing cavity between the S1–S4 helices and the TRP box (Figure 5) [109,110]. The hTRPV4 cavity is primarily composed of aromatic and polar residues, with polar side chains concentrated near the top of the transmembrane helix and aromatic rings centrally positioned [64]. This arrangement allows various ligands, both agonists and antagonists, to enter and stably bind within the cavity [69]. The identification of selective and potent TRPV4 antagonists has enhanced our understanding of the functions of TRPV4 and was crucial for advancing therapies targeting this channel [111]. The first synthetic TRPV4-selective agonist identified was 4α-PDD, which has shown efficacy in activating TRPV4 without triggering protein kinase C (PKC) activity, a common side effect in related compounds (Table 3) [112]. Site-directed mutagenesis experiments suggested that 4α-PDD binds within a pocket between the S3 and S4 helices, with key residues Y556, L584, W586, Y591, and R594 playing crucial roles in TRPV4 gating dependent on 4α-PDD [113]. Advances in TRPV4 structural studies reveal that the agonist shares the same binding site as 4α-PDD [114]. GSK1016790A, a highly potent and selective activator, effectively induces TRPV4-mediated calcium influx and enhances ciliary activity in airway epithelial cells [115]. GSK1016790A is also extensively used in pharmacological research to explore TRPV4 activation [116]. Endogenous activators, including oxidative metabolites of arachidonic acid like 5,6-epoxyeicosatrienoic acid, further demonstrate TRPV4’s therapeutic potential [117]. RN-1734 was found to be selective for TRPV4 and could thus be a valuable pharmacological tool for TRPV4 studies [106].

Recent cryo-EM structures suggest that these agonists share the same ligand-binding site as synthetic antagonists [69]. In recent years, several TRPV4 inhibitors have been developed, including HC-067047, RN-1734, RN-9893, GSK2193874, PF-05214030, GSK2798745, and GSK3491943 (Table 3) [118]. The structure shows that the HC-067047 molecule is positioned within the vanilloid pocket, surrounded by residues shared with the agonists 4α-PDD and GSK1016790A [69]. Functional assays further confirmed the importance of these residues for HC-067047 binding, as alanine substitutions at these sites significantly reduced the compound’s inhibitory effect [69]. GSK2798745 is the first TRPV4 inhibitor to undergo clinical trial evaluation aimed at treating pulmonary edema associated with congestive heart failure [119]. Early-phase trials have shown that GSK2798745 is safe and well-tolerated in humans [119,120]. The variability in efficacy among different TRPV4 modulators, such as the lower potency of 4α-PDD compared to GSK1016790A, highlights the importance of continual research on new compounds, and research into the pharmacokinetics, safety, and efficacy of these modulators will be crucial in translating these findings into clinical practice. As this review highlights the complex role of TRPV4 activation in CF, some studies highlight the potential benefits of TRPV4 activation [80,81,82,83], while others suggest that inhibiting TRPV4 could significantly reduce lung inflammation [100]. Their evidence indicates that TRPV4 exacerbates the inflammatory response in CF, contributing to tissue damage via dysregulated signaling pathways. One study mentions that TRPV4’s dual role in mechanosensation and immune regulation, termed “mechanoimmunology”, implicates it in both barrier function and immune responses [121]. This intersection of mechanical and immune functions could provide new therapeutic insights for managing CF, though the full extent of TRPV4’s involvement is yet to be understood.

**Table 3:** Small molecules of TRPV4.

**Activators**	**Chemical Structure**	**Species**	**pEC_50_ (µM)**	**Remarks**	**References**
GSK1016790A	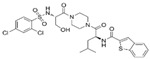	Human Mouse	8.7 7.7	A potent and selective TRPV4 activator induces Ca^2+^ influx	[113,122,123]
4α-PDD	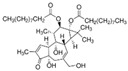	Human	6.5	Known to activate TRPV4; it increases the ciliary beat frequency	[72,123,124]
RN-1747	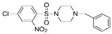	Human Mouse Rat	6.1 5.4 5.4	Selective TRPV4 agonist	[106,123]
**Inhibitors**	**Chemical Structure**	**Species**	**IC_50_ (nM)**	**Remarks**	**References**
GSK2798745	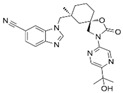	Human Rat	1.8 1.6	A potent inhibitor and selective and orally active TRPV4 ion channel	[119,120,125]
HC-067047	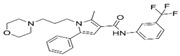	Human Rat Mouse	48 133 17	A potent and selective TRPV4 inhibitor	[126]
GSK2193874	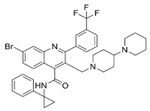	Human Rat	40 2	A potent, orally active, and selective TRPV4 blocker	[127]
PF-05214030	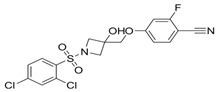	Human Rat	4 27	A TRPV4 inhibitor	[118,128]

## 7. Conclusions and Future Perspectives

TRPV4 channels play a crucial physiological role in the airway epithelium, particularly in CBF regulation and mucus clearance, which are essential processes for maintaining healthy lung function. However, current research on the specific modulation of TRPV4 in airway epithelial cells remains limited. In this review, we combined current research on TRPV4 channel modulation and its impact on the regulation of CBF in the airway epithelium. This offers valuable insights into mucus clearance mechanisms in CF and highlights the potential of TRPV4 as a therapeutic target for enhancing airway clearance in CF patients. Given the potential effects, further studies are necessary to fully elucidate the role of TRPV4 in airway physiology and its therapeutic potential. This could guide new treatments aimed at enhancing airway clearance and improving the quality of life for patients with cystic fibrosis.

## Figures and Tables

**Table 1:** TRPV gene family.

TRPV	Human Chromosomal Location	Tissue Distribution	Physiological Roles	Reference
TRPV1	17p13.2	Brain, kidney, bladder, skin, pancreas, lung macrophages, epithelial cells, T-lymphocytes	Sensing heat, pain, and inflammation	[40,51,52,53,54,55]
TRPV2	17p11.2	Heart, lung, spleen, stomach, intestine	Sensing high temperatures and mechanical stimuli	[37,56,57]
TRPV3	17p13.3	Epithelial cells, skin, tongue, nose, hair follicles	Sensing warm temperatures and being involved in hair growth	[40,58,59]
TRPV4	12q24.11	Kidney, bladder, liver, blood vessels, lungs (airway smooth muscle, epithelial cells, fibroblasts, and macrophages)	Sensing osmotic pressure, mechanical stress, and heat, maintaining organ homeostasis, including in the lungs	[8,10,13,50,54,60]
TRPV5	7q34	Kidney, placenta	Calcium homeostasis	[37,61]
TRPV6	7q34	Intestine, kidney, placenta	Calcium absorption and homeostasis	[53,61,62]

**Table 2:** Main physiological roles of TRPV4 in respiratory epithelium.

Respiratory Cell Type	TRPV4 Channel Role	References
Pulmonary artery smooth muscle cells (PASMCs)	Regulates pulmonary blood flow and vascular tone	[82,83]
Tracheal epithelial cells	Modulates immune response, mucus secretion, and inflammatory cytokine release	[72,84]
Bronchial epithelial cells	Mechanotransduction, regulation of calcium signaling, and modulation of inflammatory responses	[85,86]
Cilia of bronchial epithelial cells	Regulates ciliary beat frequency and mechanosensation	[10,87]
Bronchopulmonary sensory neurons	Indirectly modulates respiration through sensory neuron activation	[81]
Alveolar epithelial cells	Contributes to the alveoli–capillary barrier function, maintains epithelial barrier integrity, prevents edema formation, and regulates fluid balance	[49,77]

## Data Availability

Data sharing is not applicable.

## Abbreviations

Adenosine triphosphate (ATP)
Airway surface liquid (ASL)
Big conductance Ca^2+^-activated K^+^ (BK)
Calcium (Ca^2+^)
Cystic fibrosis (CF)
Cystic fibrosis transmembrane conductance regulator (CFTR)
Ciliary beat frequency (CBF)
Calcium-dependent chloride channel (CaCC)
Chronic obstructive pulmonary disease (COPD)
Epithelial sodium channels (ENac)
Magnesium (Mg^2+^)
Mucociliary clearance (MCC)
Periciliary liquid (PCL)
Potassium (K^+^)
Pulmonary artery smooth muscle cells (PASMCs)
Regulatory volume decrease (RVD)
Transmembrane helices (TMs)
Transient receptor potential vanilloid (TRPV)
Voltage sensor-like domain (VSLD)
Voltage-gated ion channels (VGICs)
Wild type (WT)
